# On enzymatic remodeling of IgG glycosylation; unique tools with broad applications

**DOI:** 10.1093/glycob/cwz085

**Published:** 2019-10-16

**Authors:** Jonathan Sjögren, Rolf Lood, Andreas Nägeli

**Affiliations:** Genovis AB, Scheelevägen 2, 223 63 Lund, Sweden

**Keywords:** Antibodies, Endoglycosidases, EndoS, Enzymatic tools/, IgG glycosylation

## Abstract

The importance of IgG glycosylation has been known for many years not only by scientists in glycobiology but also by human pathogens that have evolved specific enzymes to modify these glycans with fundamental impact on IgG function. The rise of IgG as a major therapeutic scaffold for many cancer and immunological indications combined with the availability of unique enzymes acting specifically on IgG Fc-glycans have spurred a range of applications to study this important post-translational modification on IgG. This review article introduces why the IgG glycans are of distinguished interest, gives a background on the unique enzymatic tools available to study the IgG glycans and finally presents an overview of applications utilizing these enzymes for various modifications of the IgG glycans. The applications covered include site-specific glycan transglycosylation and conjugation, analytical workflows for monoclonal antibodies and serum diagnostics. Additionally, the review looks ahead and discusses the importance of O-glycosylation for IgG3, Fc-fusion proteins and other new formats of biopharmaceuticals.

## Introduction

As the field of glycobiology continues to impact many fields of biological research, the challenges associated with analysis and study of glycoproteins have increased significantly. In recent years, the instrumentation to study glycans and glycoproteins has taken major leaps in resolution, throughput and sensitivity. However, the preparation of the often complex and diverse sample has suffered from lack of specific tools. This has stimulated innovation and new ways of using naturally occurring enzymes with glycosidase activities to study the biology of carbohydrates on proteins. During the same time, antibody-based drugs have risen and occupied major parts of the top 10 list of top-selling pharmaceuticals and according to the clinical pipeline, this class of biological drugs will continue to improve patients’ lives for many years to come (Kaplon and Reichert 2019). One of the most studied glycans are the N-glycans of human IgG for their role in therapeutic antibody drugs and the impact on the biopharmaceutical efficacy, safety and function (Rudd et al. 2001). IgG glycosylation not only impacts therapeutic antibodies but naturally also has a role in the human defense mechanisms against foreign material and invading pathogens. Pathogens that have co-evolved with humans for long periods of time have enzymes that specifically modify IgG glycosylation as a way of inactivating the antibody to circumvent the immune system and cause disease (Collin and Olsén 2003; Sjögren & Collin 2014). This review article focuses on applications of enzymes acting specifically on the important IgG glycan as biotechnological tools to study structure function relationships and selectively modify Fc glycans for remodeling using glycan substrates or other payloads, or as diagnostic tools and much more. The interest in the IgG glycans combined with the discoveries of IgG-specific endoglycosidases (as well as general exoglycosidases and endo-N-acetylgalactosaminidases) have driven new applications that expand the toolbox for analyzing and engineering the IgG (Fc-)glycans. This review aims to cover the recent advancements in this field.

### IgG glycosylation

**Fig. 1:**
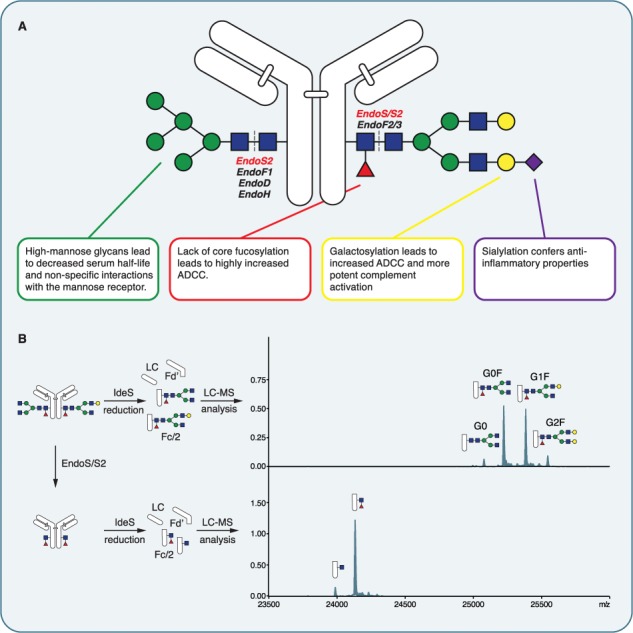
IgG Fc glycosylation. (**A**) Schematic representation of IgG glycosylation with a short summary of the main glycan features and their effects on IgG effector functions. The digestion sites for the different endoglycosidases are marked. (**B**) Hydrolysis of IgG Fc glycans by EndoS/EndoS2 analyzed by middle-level mass spectrometry. After treatment with endoglycosidase, the mAbs were cleaved with IdeS and reduced and the resulting subunits were analyzed by LC-MS. The deconvoluted spectra of the Fc/2 fragment reveals the glycosylation pattern of the mAb in the control sample (top) and demonstrates complete glycan hydrolysis after endoglycosidase treatment (bottom).

Antibodies are essential components in the human immune system and the defense against foreign material and invading pathogens. All human antibodies are glycoproteins, carrying a varying degree of glycosylation, and the functions of the glycans are being revealed for the different subtypes of antibodies (Rudd et al. 2001; Shade and Anthony 2013; Shade et al. 2019). IgGs consist of two heavy chains and two light chains that together make up two distinct parts; the antigen-binding fragment, Fab, and the crystallizable fragment, Fc. The Fab part contains the complementary-determining regions (CDRs) that interact with the antigen, and the Fc contains binding sites for Fc γ-receptors (FcγRs) and the complement system. The glycosylation site on Fc is highly conserved at position Asn297 of each heavy chain and is occupied with biantennary complex type N-glycans (Figure 1). The two glycans are located in the CH2 domain on the inner side of the horseshoe-shaped Fc. The glycan itself has been shown to be flexible and highly dynamic which may allow glycosidases to reach the glycan for modification, to expand the understanding of Fc-receptor interactions with the Fc-glycan (Barb and Prestegard 2011). In human serum, there are 33 described glycoforms present on IgG to varying degrees allowing a fine-tuning of the antibody affinities to FcγRs and thus the functions within the immune system (Pucić et al. 2011; Jennewein and Alter 2017). The FcγRs are themselves glycosylated, and their carbohydrates play a role in the differential binding to immunoglobulins (Hayes, Cosgrave, et al. 2014a; Hayes, Frostell, et al. 2014b). Also, the size of immune complexes formed through antibody binding combined with glycosylation impacts the interaction with FcγRs (Lux et al. 2013). The specific glycoforms of IgG have been associated with functional aspects of the antibody molecule, explained in part by a protein-protein and glycan–glycan interaction between the IgG and certain FcγRs (Radaev and Sun 2001; Radaev et al. 2001; Woof & Burton 2004) (Figure 1).

For example, fully sialylated IgG glycans are associated with increased anti-inflammatory properties. IgG is often sparsely sialylated, with only ca. 10% of the glycans being mono-sialylated, and an even smaller fraction di-sialylated (Quast and Lünemann 2014). Levels of IgG sialylation have however been associated with several pathologies, with a reduced sialylation degree (and galactoses) in autoimmune diseases (Parekh et al. 1985; Parekh et al. 1988; Sumar et al. 1991). A high abundance of sialic acids has thus been recognized as anti-inflammatory (Quast and Lünemann 2014), but the mechanism thereof has been elusive (Brückner et al. 2017). While the anti-inflammatory effect of sialic acids on IgG is undisputable, several mechanisms to explain the effect have been proposed (Collin and Ehlers 2013). Most data suggest a modified interaction of IgG with Fc receptors (Kaneko et al. 2006) or direct binding to DC-SIGN (Anthony et al. 2011), though the findings have been criticized (Guhr et al. 2011). For readers specifically interested in the role of sialylation on IgG, they are referred to two excellent reviews (Böhm et al. 2014; Brückner et al. 2017).

Galactosylation is another key glycosylation marker, important for the binding between IgG and C1q and in this way activation of the complement system. The same sugar also impacts the interactions between IgG and FcγRs (Nimmerjahn and Ravetch 2007). The exposure of terminal GlcNAc (so called G0 glycans) is elevated in rheumatoid arthritis and cause increased binding to mannose-binding lectin and is thought to contribute to the chronic inflammation through this pathway (Malhotra et al. 1995).

The vast majority of the IgG is fucosylated (85%) on the core GlcNAc (Mizuochi et al. 1982). The degree of IgG without fucose is highly variable between individuals and ranges between 1.3 and 19.3% of total IgG (Pucić et al. 2011). The fucose residue located at the core GlcNAc has a major impact on the interaction with FcγRIIIa (Shields et al. 2002) and afucoslyated antibodies display increased antibody-dependent cell-mediated cytotoxicity (ADCC) (Bruggeman et al. 2017) which has made this residue highly interesting for therapeutic antibodies. While some therapeutic monoclonal antibodies function through induction of apoptosis of targeted cells by binding to and blocking of vital signaling receptors, most exert their function through ADCC (Pereira et al. 2018). There are now glycoengineered antibodies in the clinic with reduced fucose levels (Ferrara et al. 2011; Beck and Reichert 2012).

High-mannose glycans are occurring on IgG in human serum in trace amounts (<0.1%), but antibodies expressed in Chinese hamster ovary (CHO) cells usually display between 1 and 10% of this incompletely processed glycan (Flynn et al. 2010). The antibodies carrying high-mannose residues have been shown to be cleared faster in humans compared to antibodies carrying other glycoforms (Goetze et al. 2011). Taken together, the Fc glycosylation profile impacts the functionality of the antibody. However, in humans not only the Fc domain is glycosylated. In studies of IgG in human serum, up to 15–25% of the antibodies carry a second glycosylated asparagine in the Fab domain in addition to the conserved Fc glycan (Bondt et al. 2014). In human serum samples, up to 37 different glycoforms have been described from Fab glycosylation sites. Glycosylated asparagine residues in the Fab are not conserved, and thus, the function of the glycan varies and may affect immunoreactivity and have an impact on affinity and avidity of the antibody as well as antibody half-life (Holland et al. 2006; Stadlmann et al. 2010). An example from biopharma is the therapeutic antibody cetuximab produced in the mouse Sp2/0 cell line containing a Fab glycosylation site at Asn88 that display a highly heterogeneous glycosylation profile including α-1,3-linked galactose residues, causing hypersensitivity in some patients (Chung et al. 2008; Jefferis 2009).

### Endoglycosidases specific for IgG

Glycosidases are defined as enzymes that catalyze the hydrolysis of glycosidic bonds in complex sugars (Bourne and Henrissat 2001). The group of enzymes can be divided into exoglycosidases acting on terminal residues or endoglycosidases acting within the glycan structure. Bacterial endoglycosidases with applications in the study of human glycoproteins and glycoforms are legio, and the majority display activity with glycoform specificity (Garbe and Collin 2012; Sjögren & Collin 2014). Classic examples include EndoH that specifically hydrolyzes high-mannose and some hybrid-type glycans (Robbins et al. 1984); EndoF family consisting of EndoF1, EndoF2 and EndoF3 all with different specificities and having a tendency not to digest all N-glycans and in some cases require protein denaturation for substrate access and glycan hydrolysis (Elder and Alexander 1982; van de Bovenkamp et al. 2019); EndoD from *Streptococcus pneumoniae* that cleaves at the chitobiose core of N-glycans (Muramatsu 1971). The applications of the classic endoglycosidases have been limited by their specificities for either unusual glycoforms or limited activity on native glycoproteins. A family of endoglycosidases with high specificity for IgG has been discovered and characterized from the genus *Streptococcus,* namely EndoS, EndoS2, EndoSe and EndoSd (Collin and Olsén 2001a; Flock et al. 2012; Sjögren et al. 2013; Shadnezhad et al. 2016) (Figure 1A). Since then, EndoS and EndoS2 have become valuable tools for antibody research, as will be discussed later in this review, while the enzymes EndoSe from *Streptococcus equi* subsp*. equi* and EndoSd from *Streptococcus dysgalactiae* subsp*. dysgalactiae* have not yet led to published applications, possibly due to an overlap in the properties compared to the other mentioned endoglycosidases.

### Endoglycosidase S and S2

The first endoglycosidase with specific activity on IgG was discovered in 2001 by Collin et al. and denoted EndoS, endoglycosidase from *Streptococcus pyogenes* (Collin and Olsén 2001a). The glycosidase activity on IgG was discovered as a shift in electrophoretic mobility of the IgG heavy chain after incubation with culture supernatants of *S. pyogenes*. The activity was determined to come from a secreted 108 kDa protein in the supernatant, and after recombinant expression, the activity was tested on a range of immunoglobulins and was found to be limited to IgG (Collin and Olsén 2001b). To date, no other substrate proteins for EndoS have been described and the observed IgG specificity has later been confirmed by detecting specific release of IgG-type glycans from human serum when incubated with EndoS (Vanderschaeghe et al. 2018). The enzymatic activity was located to the chitobiose core of the complex Fc N-glycan, and the active site of the enzyme confirmed a catalytic site belonging to the glycoside hydrolase family 18 (GH18) with glutamic acid 235 being essential for the enzymatic activity (Allhorn et al. 2008b) (Figure 1A). The hydrolysis of the IgG glycan after the core GlcNAc was shown to abolish binding to Fc receptors by surface plasmon resonance (Allhorn et al. 2008a).

A unique endoglycosidase with activity on IgG glycans was discovered in serotype M49 of *S. pyogenes* and was denoted EndoS2 (Sjögren et al. 2013). Interestingly, this enzyme is encoded in the exact same locus on the genome as EndoS but holds only 37% identity to the ndoS gene; still, EndoS2 displays a similar enzymatic activity in specifically hydrolyzing the Fc glycan of IgG. The enzyme was characterized as an endo-β-N-acetylglucosaminidase that hydrolyzed the Fc glycans of IgG with a broader glycoform specificity as compared to EndoS, where EndoS2 hydrolyzes all glycoforms including high-mannose, hybrid and complex types independent of the fucosylation status (Sjögren et al. 2015) (Figure 1A). The specificity for the Fc site was indicated both by studying the released glycans from cetuximab, where EndoS2 only released glycoforms present in the Fc domain, and the need for an intact IgG structure for enzymatic activity (Sjögren et al. 2013; Sjögren et al. 2015). The glycosidase activity of EndoS2 is demonstrated at the antibody subunit level using IdeS digestion (middle-level analysis generating Fd, LC and Fc/2 fragments) and liquid chromatography coupled to mass spectrometry and demonstrates the clear hydrolysis of glycoforms down to the core GlcNAc with or without the core fucose at the Fc/2 fragment (Figure 1B).

The crystal structure of the endoglycosidase EndoS was solved by Trastoy et al. in 2014 and revealed a V-shaped structure with five domains; glycosidase domain, leucin-rich repeat domain, hybrid Ig, carbohydrate-binding module and a three-helix bundle domain (Trastoy et al. 2013; Trastoy et al. 2014). The specific interaction between the substrate G2 glycan at the Fc domain and EndoS has recently been studied in greater detail and showed two asymmetric grooves that holds the N-glycan and several loops that guide the strict specificity of the enzyme (Trastoy et al. 2018). There is also an interaction between EndoS and the IgG molecule itself, as demonstrated by the requirement for native folding of IgG in the first paper describing the enzyme and a truncation study, which showed the necessity of the CH2 domain for glycosidase activity (Dixon et al. 2014). The crystal structure of EndoS2 was recently solved and provided structural insights into the broader glycoform specificity as the asymmetric grooves accommodated both high-mannose and complex-type glycans (Klontz et al. 2019). The interaction between EndoS2 and IgG was also studied using hydrogen-deuterium exchange mass spectrometry (HDX-MS) and showed that the carbohydrate-binding motif is involved in guiding the specificity through an interaction with the CH2 domain (Klontz et al. 2019).

The impact of EndoS in streptococcal pathogenesis has been discussed for many years, and it has been difficult to mimic the mechanisms of infection of this strict human pathogen in relevant models of infection. It has been shown that enzymatic hydrolysis of glycans from IgG using EndoS reduces opsonophagocytosis and complement activation using ex vivo experiments (Collin et al. 2002). However, a homologous knockout of the gene ndoS (encoding the protein EndoS) in the highly virulent M1T1 strain of *S. pyogenes* showed no significant contribution to virulence in mouse models of infection but showed reduced phagocytosis and bacterial killing in in vitro experiments using human cells (Sjögren et al. 2011). These hurdles were recently overcome by studying samples from patients infected with *S. pyogenes* using targeted mass spectrometry. Using this approach, the specific activity of the enzyme was studied in the natural host and it was demonstrated for the first time that the hydrolysis of the IgG glycan by EndoS occurs at the local site of infections and systemically in severe cases of streptococcal infections. Using the in vivo findings as guides, relevant conditions for in vitro models were developed and it was further demonstrated that an EndoS-knockout streptococcal strain showed significant decrease in pathogenicity during infection in mice immunized with M protein (Naegeli et al. 2019).

While our knowledge of the biological role of endoglycosidase S and S2 in host–pathogen interactions, and even more so in host–carrier interactions, is still in its infancy, more focus has been directed towards EndoS and EndoS2 as biotechnological tools. The following sections will detail our current knowledge regarding the unique endoglycosidases EndoS and EndoS2 and their broad applications within the biotechnological field.

### Different approaches for tailoring IgG Fc glycosylation

#### Genetic approaches

Serum IgG purified from a healthy individual will be made up of up to 33 different major and minor glycoforms (Wuhrer et al. 2007; Pucić et al. 2011), all with somewhat different effector function profiles. The study of the exact functions of each of these glycoforms is hampered by the difficulty in producing homogeneously glycosylated IgG with defined structures to study these changes. Furthermore, therapeutic mAbs could benefit from tailored glycosylation patterns able to elicit the correct set of effector functions suitable for their respective mode of action, and several methods of glycoengineering have thus been developed (Mastrangeli et al. 2018; Wang et al. 2019). Successful strategies for decreasing fucosylation levels have been known for a long time (Umaña et al. 1999; Yamane-Ohnuki et al. 2004) and have since been improved. For example, a “fucose switch” was included in CHO cells allowing the production of highly fucosylated immunoglobulins to be switched into afucosylated through co-expression of GDP-6-deoxy-D-lyxo-4-hexulose reductase (Roy et al. 2018). Inactivation of GDP-fucose transporters (Slc35c1) results in similar phenotypes (Chan et al. 2016), as does the usage of the decoy substrate 2-deoxy-2-fluoro-1-fucose (high-affinity binding to the fucose binding site of fucosyltransferases), with a reduced fucosylation degree on IgG (15%, compared to 94%) (Dekkers et al. 2016). Overexpression of the bacterial GDP-6-deoxy-D-talose synthetase interfering with the regular biosynthetic pathway of fucose synthesis significantly lowers the degree of fucosylation (Kelly et al. 2018). Gene manipulation of CHO cells through removal of the fucosyltransferase *fut8* similarly results in a pool of afucosylated antibodies (Chung et al. 2012). Likewise, presence of glycosidase inhibitors during production of IgG in HEK-293F cells resulted in afucosylated high-mannose structures with improved affinity to FcγRIII and significantly better ADCC activity with PBMCs (van Berkel et al. 2010). Similarly, IgG Fc sialylation can be increased by co-transfection of the IgG-expressing cells with B4GALT1 and STGALT (Dekkers et al. 2016).

Finally, with the advent of more advanced genome-editing technologies such as CRISPR/Cas9, it has become more and more feasible to design cell lines expressing mAbs with defined Fc glycan profiles (Chung, Wang, Yang, Yin, et al. 2017a; Chung, Wang, Yang, Ponce, et al. 2017b; Schulz et al. 2018). However, there is still a considerable amount of time and resources necessary for gene editing and clone selection until a stable cell line with the desired properties is generated. Furthermore, such approaches are only suitable to recombinant mAbs where expression conditions and cell lines can be changed. There is therefore still a need for methodologies to engineer protein glycosylation when the protein is already expressed and purified.

#### Step-wise enzymatic remodeling of IgG glycosylation

Trimming by exoglycosidase treatment or enzymatic addition of additional monosaccharide residues by recombinantly expressed glycosyltransferases has met with some success (Hodoniczky et al. 2005; Washburn et al. 2015), but not all glycan structures are equally accessible this way. Several fucosidases capable of hydrolyzing core α1,6-linked fucoses on intact glycoproteins have been characterized, but all of them rely on the removal of the vast majority of the glycan chain for effect (e.g. treatment with endoglycosidases) (Tsai et al. 2017). However, while hydrolysis of the core fucose has proven challenging, the addition thereof is surprisingly efficient. By generating an inactive AlfC fucosidase mutant (E274A), the hydrolase was reverted into a glycosyltransferase able to efficiently fucosylate intact N-glycoproteins including IgG in vitro (Li et al. 2017). Likewise, IgG Fc sialylation can be increased in vitro using recombinant sialyltransferases (Dekkers et al. 2016). Administration of recombinant galactosyl- and sialyltransferases in mice has even shown to increase IgG sialylation in vivo and has led to significantly reduced inflammation in an arthritis model (Pagan et al. 2018).

### Engineering IgG glycosylation by endoglycosidase mediated transglycosylation

**Fig. 2:**
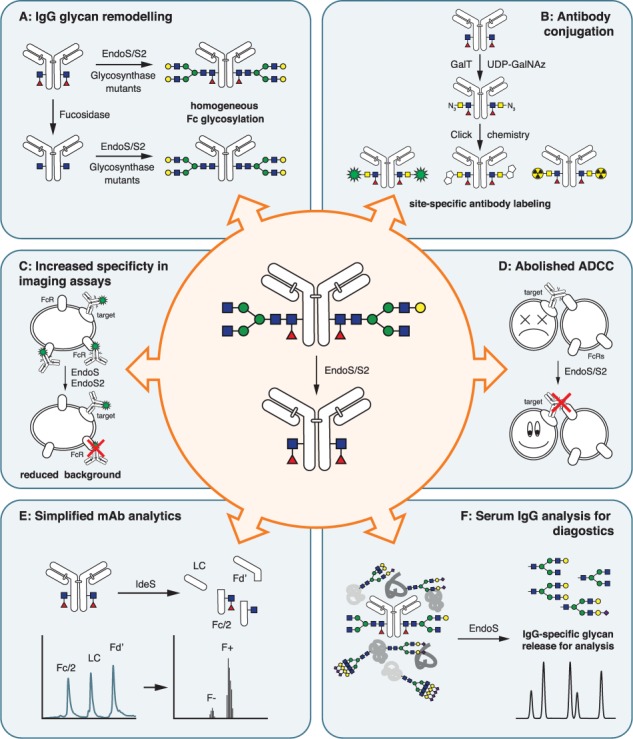
Biotechnological applications of IgG-specific endoglycosidases. Specific hydrolysis of IgG Fc glycans by endoglycosidases offers new possibilities for many different applications, such as glycan remodeling by transglycosylation (**A**), as a way to achieve homogeneous, site-specific antibody conjugates (**B**), to increase specificity in imaging assays (**C**), to abolish ADCC for mAb mode-of-action studies (**D**), to simplify analytics of mAbs (**E**) or for simplified assays for serum IgG analysis (F).

While the enzymatic approaches discussed above have met with some success in producing IgGs with tailored Fc glycans, such methods are often inefficient and less suitable to affect major changes in the glycan structures. Transglycosylation is based on the enzymatic removal of the existing glycosylation by an endoglycosidase and the subsequent re-addition of a different, homogeneous glycan structure by an endoglycosidase mutant favoring the catalysis of the reverse reaction—a so-called glycosynthase (Figure 2A). Both natural glycans, released and purified from suitable glycoproteins as well as synthetic glycan substrates, can be used for such a reaction. The reducing end is usually derivatized (e.g. by an oxazoline) to facilitate transglycosylation. This allows for complete remodeling of glycan structures, for example from high-mannose to complex-type, and gives access to homogeneously glycosylated peptides and proteins. While the methodology has been developed using a number of different substrate proteins and peptides (Li et al. 2005; Umekawa et al. 2008; Wang and Huang 2009; Schwarz et al. 2010; Huang et al. 2010; Fan et al. 2012; Lomino et al. 2013), it can also be adopted for glycoengineering of intact IgG. The early works were based on several different bacterial endoglycosidases that, while able to transfer glycans to intact IgG, had too narrow a substrate specificity to be able to transfer full-length biantennary glycans (Wei et al. 2008; Zou et al. 2011; Fan et al. 2012). The discovery of EndoS (Collin and Olsén 2001a), EndoS2 (Sjögren et al. 2013) and their respective engineered glycosynthase mutants (Huang et al. 2012; Li et al. 2016) has extended the toolbox considerably. Due to their high specificity for IgG and the biantennary complex glycans found there, these endoglycosidases are very well suited for glycoengineering IgGs via transglycosylation. Using these approaches, a number of studies have demonstrated successful remodeling of IgG Fc glycosylation with different homogeneous glycoforms (Goodfellow et al. 2012; Huang et al. 2012; Lin et al. 2015; Li et al. 2016). As discussed above, enzymatic removal of core fucosylation is difficult. However, the glycan hydrolysis step prior to transglycosylation gives much better access to the fucosylated core GlcNAc, which at this stage can be defucosylated enzymatically (Huang et al. 2012). Subsequent re-addition of the glycan by transglycosylation yields homogeneous afucosylated IgGs (Figure 2A). Even glycan structures usually not found on IgG such as tri-antennary or outer arm fucosylated structures could be transferred (Shivatare et al. 2018), giving rise to new IgG glycoforms previously not available for study. The high specificity of EndoS and EndoS2 for the IgG Fc glycan also allowed for independent, site-specific engineering of Fc and Fab glycosylation on the same molecule (Giddens et al. 2018). Endoglycosidase-mediated transglycosylation has given relatively easy access to homogeneously glycosylated mAbs with defined glycan structures which could be used to study the influence of the Fc glycan for antibody stability and function in greater detail (Wada et al. 2019).

### Site-specific antibody conjugation through modification of the Fc glycans

In addition to their role in adaptive immunity and as therapeutics, antibodies are used as detection reagents in a plethora of analytical methodologies, including western blotting, flow cytometry, ELISA and imaging techniques, to just name a few. For all of these methods, the antibodies need to be conjugated with an entity producing a measurable signal, such as a fluorophore, radioisotope or an enzyme producing some sort of detectable substrate. Furthermore, antibody–drug conjugates are gaining importance in the field of oncology. These therapeutics combine the specificity of monoclonal antibodies with the potency of conventional chemotherapeutics by coupling the toxins to a mAb to achieve a targeted delivery to the tumor tissue.

All of these applications require methodologies for conjugation of molecules to mAbs in an as robust and homogeneous way as possible without negatively affecting mAb functionality. Standard coupling techniques include chemical coupling to primary amides, carboxyl groups or the thiol groups of cysteines. However, these couplings are random and heterogeneous and usually lead to a mixed population of labeled mAbs, both in respect to number of molecules attached to each antibody as well as the attachment sites. Depending on the mAb sequence, random coupling might also affect antigen binding due to coupling to the CDRs. Site-specific conjugation can be achieved through incorporation of extra cysteine residues or unnatural amino acids with biorthogonal functionalities where a payload can be specifically coupled. However, such approaches require engineering of the antibody primary sequence and the producing cell line and are therefore only possible for recombinantly expressed mAbs and not polyclonal IgG or mAbs from hybridoma cell lines often used for research purposes.

The Fc glycan provides for a convenient conjugation point for site-specific conjugation as it is conserved in all IgGs and, due to the chemically distinct properties of carbohydrates as compared to proteins, allows for coupling specifically to the glycan part leaving the rest of the antibody untouched. The vincinal *cis* diols of certain terminal monosaccharide residues, for example, can be selectively transformed into aldehydes by mild oxidation. This allows then for site-specific coupling using hydrazine or oxime chemistry. However, not all monosaccharides usually found in Fc *N*-glycans contain vincinal *cis* diols and not all residues that do, oxidize readily. To get a more homogeneous labeling, generation of more homogeneous glycan structures is necessary, either through trimming (Zuberbühler et al. 2012) or extension (Zhou et al. 2014). Endoglycosidase-mediated glycan trimming might constitute a third and easy way to remove glycan heterogeneity for generation of homogeneous antibody conjugates by coupling to an oxidized core fucose.

Biorthogonal chemistries such as copper-free click chemistry can be introduced site specifically into glycosylated proteins by modifying their glycans with unnatural, azide-modified sugar residues. However, similar to the oxidation/hydrazine method described above, the natural heterogeneity of Fc glycans poses challenges for homogeneous antibody labeling. Several solutions have been proposed: in vitro galactosylation followed by enzymatic transfer of azide-activated sialic acid was used to produce mAbs suitable for site-specific coupling using click chemistry (Li et al. 2014). The same structures could also be achieved by endoglycosidase-mediated transglycosylation of a glycan decorated with azide-modified, terminal sialic acids (Tang et al. 2016). Azide-activated N-acetylgalactosamine (GalNAz) can also be transferred to terminal GlcNAc residues by a mutant galactosyltransferase (Ramakrishnan and Qasba 2002). To generate these terminal GlcNAc residues, the mAbs can be treated with β-galactosidase to generate homogeneous G0 glycoforms (Zeglis et al. 2013), or the glycan can be removed using endoglycosidase EndoS or EndoS2. This leads to a single GlcNAc residue left on the protein which can serve as a substrate for GalT (Figure 2B). In this way, homogeneous site-specifically coupled antibody conjugates can be generated (van Geel et al. 2015; Toftevall et al. 2019). This methodology also has the added benefit that the removal of the Fc glycan impairs binding of the antibody to Fc receptor and complement, leading to reduced background in assays such as flow cytometry or imaging (Gao et al. 2015) and potentially reduced off-target effects of ADCs.

### Impairing Fc receptor interactions

When IgGs are used as detection reagents in more complex cellular systems, as might be the case in flow cytometry, immunofluorescence imaging of cells or tissue samples or in vivo imaging, they can interact not only with their specific antigen target but also with natural occurring proteins including Fc receptors and the complement system. Therefore, these antigen-independent interactions can cause increased background or in the worst case false positives. As removal of the Fc glycan greatly impairs IgG interactions with Fc receptors (Allhorn et al. 2008a; Lux et al. 2013) and complement (Collin et al. 2002), enzymatic Fc deglycosylation of IgG used for detection could improve the specificity of many antibody-based assays routinely used in research or diagnostics (Figure 2C) and would abolish the need for isotype controls and gating strategies used nowadays to manage background levels (Andersen et al. 2016). Such an approach has been successfully employed to improve the analytical sensitivity and specificity when studying lymph node metastasis in vivo using near-infrared fluorescence molecular imaging (Gao et al. 2015).

### IgG-specific endoglycosidases as tools for analysis of mAbs

Monoclonal antibodies (mAbs) are important biotherapeutics and are successfully used to treat an increasing number of diseases such as cancer and inflammatory diseases. Compared to conventional small molecule drugs, these macromolecules are much larger and much more heterogeneous. This poses analytical challenges as a large number of different quality attributes have to be analyzed and quantified to ensure a robust manufacturing of safe and potent biopharmaceuticals. The current gold standard for characterization of protein pharmaceuticals is bottom-up peptide mapping by LC-MS. While this is a very powerful technique able to produce data on many different post-translational modifications, it is also very time- and resource-consuming. With the development of more sensitive and more accurate mass spectrometry instrumentation, top-down or middle-down workflows are getting more and more popular. These entail the analysis of intact mAbs (top-down) or antibody fragments (middle-down, Figure 1B) using high-resolution mass spectrometry. While these workflows are much faster and yield easier interpretable data, they also suffer from drawbacks. As many modifications and critical quality attributes are measured on the same molecule, it is not always possible to identify and/or locate the specific modifications. For example, the natural heterogeneity of the Fc glycosylation might mask other important modifications. The IgG-specific endoglycosidases EndoS and EndoS2 provide a quick and simple way to remove this heterogeneity and thereby simplify the analysis of other critical quality attributes.

Methionine residues on proteins oxidize easily, a modification that may have detrimental effects on mAb functions such as antigen binding or stability (Dashivets et al. 2016; Mo et al. 2016). Analysis of mAb oxidation can be achieved by peptide mapping, which is time-consuming, or LC-based separation and quantification of oxidized and non-oxidized mAbs or mAb subunits. MS-based approaches at the intact or middle level suffer from difficulties in resolving isobaric structures. For example, a fucosylated and oxidized mAb has the same mass as an afucosylated one containing an additional galactose. Removal of Fc glycosylation by endoglycosidase treatment reduces complexity and allows for easier analysis by LC-MS (Leblanc et al. 2014; Sokolowska et al. 2017). Similarly, glycation—the spontaneous reaction of reducing sugars with primary amines on the protein—leads to the same +162 Da mass difference as between the G0 and G1 or the G1 and the G2 glycoforms and these structures can therefore not easily be assigned by mass spectrometry. EndoS digestion rapidly and specifically removes the Fc glycan to allow for analysis of glycation by MS at the intact or middle level (Goetze et al. 2012; Mo et al. 2018).

The Fc glycosylation pattern itself is a critical quality attribute, and methods to analyze it range from analysis of released glycans by HILIC-HPLC or CE to analysis by MS at the peptide, middle or intact level (Reusch, Haberger, Falck, et al. 2015a; Reusch, Haberger, Maier, et al. 2015b). However, not all features of the Fc glycosylation are equally important, which is why methods for quick and robust analysis of important glycan structures are of interest. Core fucosylation of mAbs is especially important due to its influence on ADCC and therefore has to be monitored carefully. Even minor decreases in mAb fucosylation levels can have a large impact on the antibody’s activity profile (Thomann et al. 2016). While core fucosylation can be determined from the results of any standard IgG N-glycan analysis method, the afucosylation signals will be spread over all the different glycoforms and therefore hard to quantify. The endoglycosidases cleave in the chitobiose core and leave the potentially fucosylated GlcNAc attached to the protein. Therefore, endoglycosidase digestion of mAbs will remove the glycoform heterogeneity and allow for easy quantification of the only two remaining glycoforms (GlcNAc and GlcNAc-Fuc) by middle-level MS analysis (Figure 2E) (Liu and Zang 2016; Upton et al. 2016). Using this approach also answers the question of site-occupancy of the glycans at the Fc domain in a single experiment. Similar approaches were also successful on a proteome-wide level using the protein-unspecific endoglycosidases EndoF1–3 in combination with trypsin (Jia et al. 2009; Ma et al. 2015; Ma et al. 2018).

### Simplified analysis of serum IgG glycosylation for diagnostics

As the structure of the Fc glycan influences IgG effector functions, it might not be surprising that changes in IgG glycosylation profiles correlate with different disease states (Parekh et al. 1985; Tomana et al. 1992; Go et al. 1994; Malhotra et al. 1995; Saldova et al. 2008; Novokmet et al. 2014). Specifically, low galactosylation of serum IgG is a hallmark of many inflammatory diseases including rheumatoid arthritis, systemic lupus erythematosus, inflammatory bowel disease and several cancers. Despite its potential as a diagnostic maker, IgG galactosylation is not routinely analyzed in the clinics, largely due to the lack of an easy, robust and cheap way of performing these analyses. A standard workflow for analysis of serum IgG Fc glycosylation would entail the purification of the antibodies by affinity capture, release of the N-glycans by PNGase F and analysis of the released glycans by HILIC-LC, CE or MS (Huffman et al. 2014). These approaches are too time-consuming for clinical practice and suffer from a lack of specificity for Fc glycans leading to interfering signals from Fab glycosylation as well as other glycoprotein impurities from the affinity capture step. EndoS, due to its protein substrate specificity, provides a facile way to release IgG Fc glycans specifically, directly from a complex sample (Figure 2F). These released glycans could then be labeled and analyzed by CE to easily and rapidly assess IgG Fc galactosylation in patient serum (Vanderschaeghe et al. 2018). This much simpler assay is high-throughput and automatable and might be robust enough to implement in clinical practice.

### IgG glycan hydrolysis abolishes ADCC

**Fig. 3:**
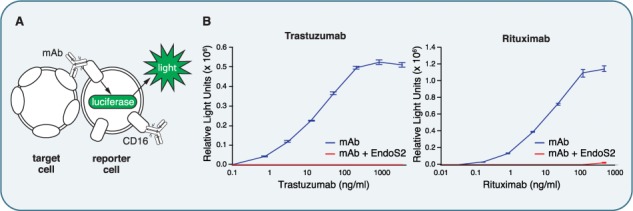
IgG glycan hydrolysis abolishes ADCC. (**A**) Illustration of the iLite® ADCC assay (Svar Life Science). Target cells expressing the antigen are combined with the mAb of interest and reporter cells expressing FcγRIII (CD16). The reporter cells further express a luciferase induced by CD16 signaling, leading to an easy read-out for activation of ADCC. (**B**) ADCC signal of the monoclonal antibodies trastuzumab (left) and rituximab (right) as determined by the iLite® ADCC assay. The signals elicited by the untreated, fully glycosylated mAbs is shown in blue, the signal from the endoglycosidase-treated mAbs in red. Note the almost complete abolishment of CD16 signaling upon IgG glycan hydrolysis.

Antibody-dependent cell-mediated cytotoxicity (ADCC) involves the interaction between the IgG Fc and the low-affinity FcγRIII receptor (CD16) on immune cells such as NK cells. This triggers a signaling cascade in the immune cell leading to the release of cytotoxic factors and the death of the target cell. As this receptor interaction is strongly influenced by the IgG Fc glycan (Subedi and Barb 2015; Subedi and Barb 2016), it can be impaired by IgG glycan hydrolysis using endoglycosidases EndoS or EndoS2. Treatment of a mAb with EndoS2 leads to the fast removal of the Fc glycan (Figure 1B). This completely abrogates CD16 signaling—a proxy for ADCC—in a reporter cell assay (Figure 3). As not all FcγR interactions are equally affected by EndoS-mediated glycan hydrolysis (Lux et al. 2013; Kao et al. 2015), the endoglycosidases provide a potentially valuable tool for dissecting the mode of action of new mAb therapeutics during preclinical development. The ability for EndoS to block certain IgG effector functions has also led to a wide range of studies investigating its potential as an IgG-specific immunosuppressant, and the protein has shown promise in an experimental animal model of many different autoimmune diseases (Nandakumar et al. 2007; Collin et al. 2008; Albert et al. 2008; Allhorn et al. 2010; van Timmeren et al. 2010; Yang et al. 2010; Lood et al. 2012).

### Effect of O-glycosylation on immunoglobulins and IgG fusion proteins

Compared to the well-known and well-studied N-glycans, O-glycans have historically received much less attention. However, their presence in the hinge region of immunoglobulin and immunoglobulin-fusion proteins has led to an increased interest from antibody researchers to be able to study these glycans and their role more. Initiated by the addition of an N-acetylgalactosamine on Ser/Thr (Tn antigen), O-glycans are expanded through a variety of glycosyltransferases in the Golgi (Zhang et al. 2016). O-glycans can be distinguished by their core structure, of which four are commonly found in human proteins (core 1–4), but a total of eight are recognized in different species (Hattrup and Gendler 2008). Due to the heterogeneity and complexity of the O-glycans, an O-glycosidase counterpart to the amidase PNGaseF is lacking, hampering the rapid progress in the understanding of the importance of O-glycans. However, during the last two decades there has been a surge in research regarding the prevalence and relevance of O-glycans, as well as the development of novel methods for the study thereof. Here, we will discuss recent aspects of how O-glycans impact disease and diagnostics, as well as methods for analysis.

#### Mucin O-glycosylation in carcinoma—a diagnostic marker

One of the most O-glycosylated protein families in our body are the mucins; hence, all O-glycan core structures are based on mucins (Bergstrom and Xia 2013), with core 1 (Gal-GalNAc-S/T) being most prevalent in human tissues (Byrd and Bresalier 2004). Core 1 glycan motifs have been demonstrated to significantly limit development of colitis and infiltration of bacteria (Fu et al. 2011), while other core glycan structures (e.g. cores 2 and 4) similarly play a role in affecting the permeability of the intestines, but to a lesser extent (Stone et al. 2009).

In several epithelial cell carcinomas, MUC1 is displaying an aberrant O-glycosylation that is now recognized as a diagnostic biomarker (Stuchlová Horynová et al. 2013). The glycosylation pattern of mucin is often changed from branched core 2 glycans to truncated core 1, with a 2-fold increase in O-glycan density (e.g. higher abundancy of occupancy) (Stuchlová Horynová et al. 2013). Antibodies directed towards such epitopes on mucin are protecting and attempts to identify means to improve such protective antibodies are underway (Rangappa et al. 2016). Aberrant O-glycosylation is however not unique for MUC1, but is a common phenomenon seen in many tumors, where terminal GalNAcs (e.g. Tn antigens) are overexpressed (Madsen et al. 2012). Due to this abundance, much focus has been directed towards targeting such antigens through cancer immunotherapy. The epitope is inducing a strong antibody response but has a limited presentation to CD8+ T cells needed for efficient tumor removal (Madsen et al. 2012).

#### Immunoglobulin O-glycosylation

O-glycosylation in immunoglobulins has been recognized in particular for IgA, being the most abundant O-glycoprotein in plasma, where the hinge region is covered by O-glycans to limit access for bacterial proteases. The glycans, mainly corresponding to di-sialylated core 1 O-glycans number 3–5 per heavy chain (Novak et al. 2000). However, a change from sialylated T antigens (NeuAc-Gal-GalNAc) to single terminal GalNAcs (Tn antigen) is often seen in IgA nephropathy (Stuchlová Horynová et al. 2013). However, early analyses by Smith et al. using lectin ELISAs demonstrated an antigen-dependent variation of O-glycoforms on IgA_1_; variations that were anticipated to be identified in any individual and thus not be a specific marker for disease (Smith et al. 2006). Using a similar setting, Satake et al. demonstrated a significant increased abundance of Tn antigens on IgA in IgAN (IgA nephropathy) patients. However, for polymeric IgA_1_ such correlation could not be observed (Satake et al. 2014). More recently, lectin chromatography has been coupled to mass spectrometry for a higher resolution of the O-glycoform pool. The authors concluded that IgA_1_ from IgAN and healthy individuals are comparable in their O-glycoforms; both having a low degree of Tn and STn antigens (sialylated Tn antigen) s (Lehoux and Ju 2017). It should however be noted that all of the above described methods are based on lectin purification, which may confer bias in the O-glycan pool studied. Opposite to epithelial carcinoma where antibodies directed towards the aberrant O-glycans are beneficial, antibodies targeting the IgA O-glycan epitope will enhance the disease progression.

**Fig. 4:**
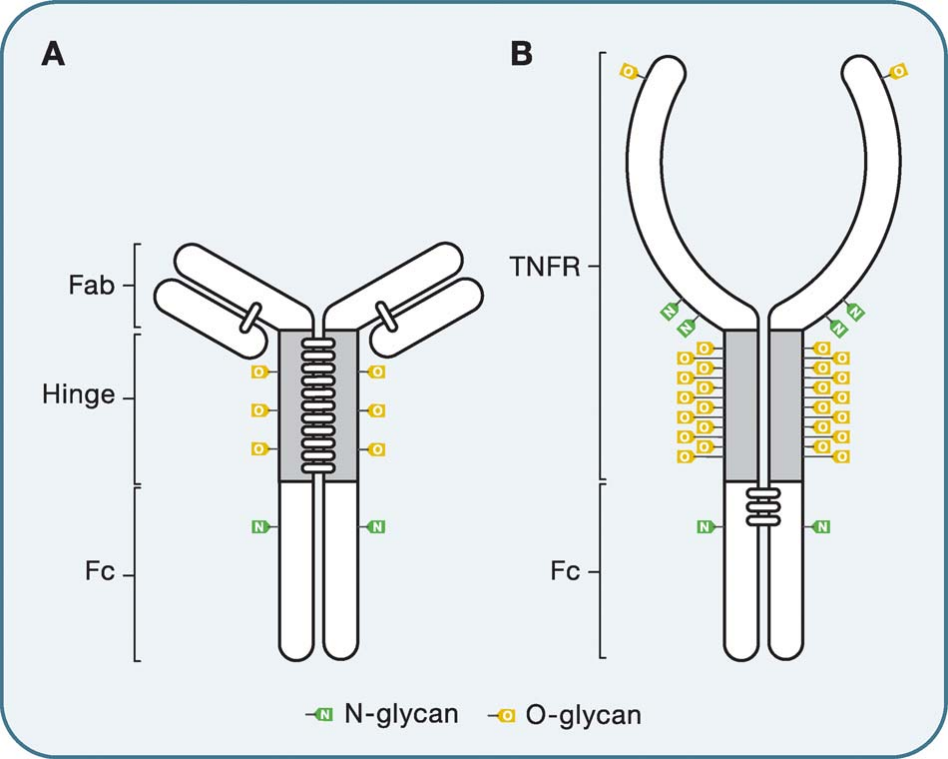
O-glycosylation of immunoglobulin G and Ig-fusion proteins. Schematic representation of O- and N-linked glycosylation pattern of human immunoglobulin (Ig) G3 (**A**) and the TNFαRII–IgG-Fc fusion protein etanercept (**B**). Both glycoproteins have several possible O-glycosylation sites, as well as N-glycan(s).

While IgG historically has been considered to only contain N-glycans (conserved in Fc, and to a lower frequency in the Fab region), recent studies have been demonstrating O-glycosylation of IgG3 (Plomp et al. 2015). IgG3 only constitutes ca. 8% of circulating IgG in the serum and is distinct due to its elongated hinge region (Figure 4A). Using nanoLC-ESI-IT-MS/MS partial O-glycosylation of the threonines in the hinge could be detected, with an occupancy rate of ca. 10% per site. The glycans were identified as mainly disialylated core 1 glycans, with a small fraction containing GlcNAc. The impact of these glycans and regulation in disease remains to be elucidated. Several variants of N-glycans could be identified on IgG1, IgG2 and IgG4, but no O-glycans were detected in these subclasses (Plomp et al. 2017), in concurrence with the theory that O-glycans mainly are present in prolonged hinge regions.

During recent years, there has been a significant increase in the development of biologics; both therapeutic monoclonal antibodies, fusion proteins with an Fc-domain and new more complex formats with two or more different Fab fragments on the same Fc domain (Grilo and Mantalaris 2019). Fc-fusion protein and complex multimodal constructs are often decorated with several O-glycans. For example, etanercept is a fusion protein consisting of the Fc-domain of IgG1 and the extracellular domain of the TNFα receptor (TNFαRII) with a long, heavily O-glycosylated region connecting the two (Figure 4B) (Montacir et al. 2018). Several studies have attempted to study glycan composition and site occupancy of such highly complex substrates using ESI-MS and MALDI-MS (Houel et al. 2014), but the complexity and demand for high-end instruments have limited studies of O-glycans on biologics.

#### New tools for analysis of O-glycans

With the increased understanding that O-glycans play a pivotal role in health, the need to be able to study both composition and site occupancy of O-glycans has been raised. Further, for the usage of heavily (O-)glycosylated vaccines and biopharmaceuticals, it is critical to be able to demonstrate and characterize the prevalence of O-glycans (Bagdonaite and Wandall 2018). Commonly, such characterizations have been performed using β-elimination techniques (Fukuda 2001), a method that continuously is being improved in order to facilitate high-throughput screening (Kotsias et al. 2019). Still, the usage of toxic chemicals is not preferential, and the usage of enzymatic release of glycans has been suggested. Existing tools, e.g. endo-N-acetylgalactosaminidases, lack the broad specificity of their corresponding N-glycan amidase (PNGaseF) and will only release asialylated core 1 and 3 glycans, not other core structures or extended cores (Marion et al. 2009). Due to the structural conformation of O-glycans, most commercial endo-O-glycosidases require denaturing conditions for their removal. However, removal of O-glycans under native conditions can be achieved, allowing for the characterization of O-glycans on the IgG-fusion protein etanercept (Onigman et al. 2019), and the biological relevance thereof.

Besides the removal of O-glycans, other O-glycan related hydrolases have gained interest during the last decade, including proteases depending on O-glycan binding for proteolytic activity on the glycoprotein (Abdullah et al. 1991; Abdullah et al. 1992; Noach et al. 2017). With the advent of OpeRATOR (Genovis AB), a mucin-type O-glycan-dependent protease hydrolyzing directly N-terminal to O-glycosylated serine or threonine residues, site-specific analysis of O-glycans and glycan composition has been facilitated. Examples include a chemoenzymatic method for specific O-glycopeptide enrichment, modification and analysis using LC-MS that have been applied to reveal more than 8-fold new O-glycosylation sites on several glycoproteins and in Zika viruses (Yang et al. 2018a). A similar OpeRATOR-based methodology has been developed to reveal thousands of new O-glycosylation sites in human kidney tissues, T cells and human serum and indicating a potential role as a diagnostic methodology to differentiate between tumor and normal kidney tissues (Yang et al. 2018b). Further, enrichment of mucin-type O-glycopeptides using GlycOCATCH (Genovis AB) further improves the capabilities to study O-glycans and may ultimately lead to an improved understanding of the biological function of O-glycans. Another example of a tool with applications for mucin-type O-glycans is the recently discovered secreted protease of C1 esterase inhibitor, StcE, from *Escherichia coli* (Malaker et al. 2019). StcE binds to a recognition motif on mucins GPT*PSAA (* = sialyl T antigen, among others) and hydrolyzes the protein backbone. The applications include selective mucin proteolysis to study the contribution of O-glycosylated mucin structures and their function in the immune system, as exemplified in by Malaker *et al.* in the case of Siglec-7 and Siglec-9. Taken together, a broad acting O-glycosidase is still a much desired tool in the field, but the recent discoveries of OpeRATOR and StcE, combined with LC-MS, have enabled new workflows to study mucin-type O-glycans and their role in biology and on therapeutic proteins.

## Summary

The field of antibody research, development and therapeutic usage is rapidly expanding, and with that the demand for new tools. Glycans play a pivotal role in the effector functions of IgG, and as such they deserve special attention. Taking advantage of bacterial-host co-evolution, several glycosidases have been identified and commercialized in order to improve our abilities to study the impact of glycans on IgG function. Notable among those are the IgG-specific endoglycosidases EndoS and EndoS2 having shown great promise in the various applications discussed in this review. With the advent of novel glycosidases acting upon IgG our abilities to further understand, these key glycoproteins have improved. The unique enzymatic tools for glycans have led to new areas of exciting research, but there are still many questions remaining to be answered and novel tools to be discovered. We are eagerly looking forward to further advancements within the field of antibody glycosylation.

## Conflict of interest statement

All authors are employees at Genovis that provides enzymatic reagents for biotech use including some of the enzymes described here. A.N. and J.S. are shareholders of Genovis, and J.S. is listed as inventor of the patent related to the EndoS2 enzyme.

## Abbreviations

ADCC, antibody-dependent cell-mediated cytotoxicity; CDR, complementary-determining region; CHO, Chinese hamster ovary; GalNAz, azide-activated N-acetylgalactosamine; FcγR, Fc γ-receptor; HDX-MS, hydrogen-deuterium exchange mass spectrometry; mAbs, monoclonal antibodies
